# Supplementary material for: A UMLS-based spell checker for natural language processing in vaccine safety
Source: BMC Med Inform Decis Mak. 2007 Feb 12;7:3. doi: 10.1186/1472-6947-7-3 (PMC1805499; doi:10.1186/1472-6947-7-3)
Supplement: Additional file 13 — RAP application directory. Sets up directory for RAP (RDF application for PHP) source code [file 1472-6947-7-3-S13.gz › rap/doc/phpdoc/errors.html]

xml version="1.0" encoding="iso-8859-1"?


phpDocumentor Parser Errors and Warnings


Post-parsing  
ATOM\_RES.php  
Blanknode.php  
constants.php  
Dataset.php  
DatasetDb.php  
DatasetMem.php  
DatasetP.php  
DBase.php  
DbModel.php  
DbStore.php  
DC.php  
DC\_C.php  
DC\_RES.php  
FindIterator.php  
FOAF.php  
FOAF\_C.php  
FOAF\_RES.php  
GRDDLParser.php  
InfModelB.php  
InfModelF.php  
InfModelP.php  
InfRule.php  
InfStatement.php  
Literal.php  
MemModel.php  
Model.php  
ModelP.php  
N3Parser.php  
N3Serializer.php  
NamedGraphDb.php  
NamedGraphMem.php  
Node.php  
NTripleSerializer.php  
Object.php  
OntModelP.php  
OWL.php  
OWL\_C.php  
OWL\_RES.php  
Quad.php  
RDF.php  
RdfAPI.php  
RdfParser.php  
RDFS.php  
RdfSerializer.php  
RDFS\_C.php  
RDFS\_RES.php  
RdfUtil.php  
RDF\_C.php  
RDF\_RES.php  
RDQL.php  
RdqlDbEngine.php  
RdqlEngine.php  
RdqlMemEngine.php  
RdqlParser.php  
RdqlResultIterator.php  
ResAlt.php  
ResBag.php  
ResContainer.php  
ResList.php  
ResLiteral.php  
ResModel.php  
ResModelP.php  
Resource.php  
ResProperty.php  
ResResource.php  
ResSeq.php  
RSS.php  
RSS\_C.php  
RSS\_RES.php  
Statement.php  
StatementIterator.php  
SyntaxGRDDL.php  
SyntaxN3.php  
SyntaxRDF.php  
TriXParser.php  
TriXSerializer.php  
Utility.php  
VCARD.php  
VCARD\_C.php  
VCARD\_RES.php  
Vocabulary.php  
VocabularyClass.php  
VocabularyRes.php  


# ATOM\_C.php

## Warnings:

  
**Warning on line 20** - no @package tag was used in a DocBlock for class ATOM  
**Warning on line 210** - File "c:\cvs\rdfapi-php\api\vocabulary\ATOM\_C.php" has no page-level DocBlock, use @package in the first DocBlock to create one  


# ATOM\_RES.php

## Warnings:

  
**Warning on line 20** - no @package tag was used in a DocBlock for class ATOM\_RES  
**Warning on line 210** - File "c:\cvs\rdfapi-php\api\vocabulary\ATOM\_RES.php" has no page-level DocBlock, use @package in the first DocBlock to create one  


# Blanknode.php

## Warnings:

  
**Warning on line 28** - DocBlock would be page-level, but precedes class "BlankNode", use another DocBlock to document the file  
**Warning on line 28** - Unknown tag "@authors" used  


# constants.php

## Warnings:

  
**Warning on line 412** - File "c:\cvs\rdfapi-php\api\constants.php" has no page-level DocBlock, use @package in the first DocBlock to create one  


# Dataset.php

## Warnings:

  
**Warning on line 21** - DocBlock would be page-level, but precedes class "Dataset", use another DocBlock to document the file  

## Errors:

  
**Error on line 20** - unmatched ol or ul tag in DocBlock, parsing will be incorrect  
**Error on line 20** - Unclosed list tag in DocBlock, parsing will be incorrect  


# DatasetDb.php

## Warnings:

  
**Warning on line 19** - Page-level DocBlock precedes "require\_once DatasetDb.php", use another DocBlock to document the source element  
**Warning on line 22** - no @package tag was used in a DocBlock for class DatasetDb  


# DatasetMem.php

## Warnings:

  
**Warning on line 22** - DocBlock would be page-level, but precedes class "DatasetMem", use another DocBlock to document the file  

## Errors:

  
**Error on line 21** - unmatched ol or ul tag in DocBlock, parsing will be incorrect  
**Error on line 21** - Unclosed list tag in DocBlock, parsing will be incorrect  


# DatasetP.php

## Warnings:

  
**Warning on line 27** - File "c:\cvs\rdfapi-php\api\dataset\DatasetP.php" has no page-level DocBlock, use @package in the first DocBlock to create one  


# DBase.php

## Warnings:

  
**Warning on line 18** - File "c:\cvs\rdfapi-php\api\model\DBase.php" has no page-level DocBlock, use @package in the first DocBlock to create one  


# DbModel.php

## Warnings:

  
**Warning on line 31** - DocBlock would be page-level, but precedes class "DbModel", use another DocBlock to document the file  
**Warning on line 299** - Unknown tag "@throw" used  
**Warning on line 840** - Unknown tag "@throw" used  

## Errors:

  
**Error on line 31** - unmatched ol or ul tag in DocBlock, parsing will be incorrect  
**Error on line 31** - Text cannot be outside of li tag in a DocBlock list, parsing will be incorrect  
**Error on line 31** - Unclosed list tag in DocBlock, parsing will be incorrect  


# DbStore.php

## Warnings:

  
**Warning on line 31** - DocBlock would be page-level, but precedes class "DbStore", use another DocBlock to document the file  

## Errors:

  
**Error on line 31** - Unclosed list tag in DocBlock, parsing will be incorrect  
**Error on line 31** - unmatched ol or ul tag in DocBlock, parsing will be incorrect  


# DC.php

## Warnings:

  
**Warning on line 85** - File "c:\cvs\rdfapi-php\api\vocabulary\DC.php" has no page-level DocBlock, use @package in the first DocBlock to create one  


# DC\_C.php

## Warnings:

  
**Warning on line 23** - no @package tag was used in a DocBlock for class DC  
**Warning on line 494** - File "c:\cvs\rdfapi-php\api\vocabulary\DC\_C.php" has no page-level DocBlock, use @package in the first DocBlock to create one  


# DC\_RES.php

## Warnings:

  
**Warning on line 22** - no @package tag was used in a DocBlock for class DC\_RES  
**Warning on line 493** - File "c:\cvs\rdfapi-php\api\vocabulary\DC\_RES.php" has no page-level DocBlock, use @package in the first DocBlock to create one  


# FindIterator.php

## Warnings:

  
**Warning on line 24** - DocBlock would be page-level, but precedes class "FindIterator", use another DocBlock to document the file  


# FOAF.php

## Warnings:

  
**Warning on line 90** - File "c:\cvs\rdfapi-php\api\vocabulary\FOAF.php" has no page-level DocBlock, use @package in the first DocBlock to create one  


# FOAF\_C.php

## Warnings:

  
**Warning on line 22** - no @package tag was used in a DocBlock for class FOAF  
**Warning on line 407** - File "c:\cvs\rdfapi-php\api\vocabulary\FOAF\_C.php" has no page-level DocBlock, use @package in the first DocBlock to create one  


# FOAF\_RES.php

## Warnings:

  
**Warning on line 21** - no @package tag was used in a DocBlock for class FOAF\_RES  
**Warning on line 404** - File "c:\cvs\rdfapi-php\api\vocabulary\FOAF\_RES.php" has no page-level DocBlock, use @package in the first DocBlock to create one  


# GRDDLParser.php

## Warnings:

  
**Warning on line 23** - DocBlock would be page-level, but precedes class "GRDDLParser", use another DocBlock to document the file  


# InfModelB.php

## Warnings:

  
**Warning on line 30** - DocBlock would be page-level, but precedes class "InfModelB", use another DocBlock to document the file  
**Warning on line 393** - Unknown tag "@throw" used  


# InfModelF.php

## Warnings:

  
**Warning on line 29** - DocBlock would be page-level, but precedes class "InfModelF", use another DocBlock to document the file  


# InfModelP.php

## Warnings:

  
**Warning on line 20** - File "c:\cvs\rdfapi-php\api\infModel\InfModelP.php" has no page-level DocBlock, use @package in the first DocBlock to create one  


# InfRule.php

## Warnings:

  
**Warning on line 27** - DocBlock would be page-level, but precedes class "InfRule", use another DocBlock to document the file  

## Errors:

  
**Error on line 26** - unmatched ol or ul tag in DocBlock, parsing will be incorrect  
**Error on line 26** - Unclosed list tag in DocBlock, parsing will be incorrect  


# InfStatement.php

## Warnings:

  
**Warning on line 16** - DocBlock would be page-level, but precedes class "InfStatement", use another DocBlock to document the file  

## Errors:

  
**Error on line 16** - Unclosed list tag in DocBlock, parsing will be incorrect  
**Error on line 16** - unmatched ol or ul tag in DocBlock, parsing will be incorrect  


# Literal.php

## Warnings:

  
**Warning on line 25** - DocBlock would be page-level, but precedes class "Literal", use another DocBlock to document the file  

## Errors:

  
**Error on line 25** - Unclosed list tag in DocBlock, parsing will be incorrect  
**Error on line 25** - unmatched ol or ul tag in DocBlock, parsing will be incorrect  


# MemModel.php

## Warnings:

  
**Warning on line 55** - DocBlock would be page-level, but precedes class "MemModel", use another DocBlock to document the file  
**Warning on line 356** - Unknown tag "@throw" used  

## Errors:

  
**Error on line 55** - unmatched ol or ul tag in DocBlock, parsing will be incorrect  
**Error on line 55** - Unclosed list tag in DocBlock, parsing will be incorrect  


# Model.php

## Warnings:

  
**Warning on line 35** - DocBlock would be page-level, but precedes class "Model", use another DocBlock to document the file  

## Errors:

  
**Error on line 35** - Unclosed list tag in DocBlock, parsing will be incorrect  
**Error on line 35** - unmatched ol or ul tag in DocBlock, parsing will be incorrect  


# ModelP.php

## Warnings:

  
**Warning on line 26** - File "c:\cvs\rdfapi-php\api\model\ModelP.php" has no page-level DocBlock, use @package in the first DocBlock to create one  


# N3Parser.php

## Warnings:

  
**Warning on line 60** - DocBlock would be page-level, but precedes class "N3Parser", use another DocBlock to document the file  
**Warning on line 658** - Unknown tag "@acces" used  


# N3Serializer.php

## Warnings:

  
**Warning on line 42** - Page-level DocBlock precedes "define MAGIC\_STRING", use another DocBlock to document the source element  
**Warning on line 44** - no @package tag was used in a DocBlock for class N3Serializer  

## Errors:

  
**Error on line 191** - DocBlock has multiple @access tags, illegal. ignoring additional tag "@access public"  


# NamedGraphDb.php

## Warnings:

  
**Warning on line 20** - DocBlock would be page-level, but precedes class "NamedGraphDb", use another DocBlock to document the file  

## Errors:

  
**Error on line 19** - Unclosed list tag in DocBlock, parsing will be incorrect  
**Error on line 19** - unmatched ol or ul tag in DocBlock, parsing will be incorrect  


# NamedGraphMem.php

## Warnings:

  
**Warning on line 19** - DocBlock would be page-level, but precedes class "NamedGraphMem", use another DocBlock to document the file  

## Errors:

  
**Error on line 18** - Unclosed list tag in DocBlock, parsing will be incorrect  
**Error on line 18** - unmatched ol or ul tag in DocBlock, parsing will be incorrect  


# Node.php

## Warnings:

  
**Warning on line 24** - DocBlock would be page-level, but precedes class "Node", use another DocBlock to document the file  


# NTripleSerializer.php

## Warnings:

  
**Warning on line 27** - DocBlock would be page-level, but precedes class "NTripleSerializer", use another DocBlock to document the file  

## Errors:

  
**Error on line 103** - DocBlock has multiple @access tags, illegal. ignoring additional tag "@access public"  


# Object.php

## Warnings:

  
**Warning on line 23** - DocBlock would be page-level, but precedes class "Object", use another DocBlock to document the file  


# OntModelP.php

## Warnings:

  
**Warning on line 16** - Page-level DocBlock precedes "include\_once OntModelP.php", use another DocBlock to document the source element  


# OWL.php

## Warnings:

  
**Warning on line 75** - File "c:\cvs\rdfapi-php\api\vocabulary\OWL.php" has no page-level DocBlock, use @package in the first DocBlock to create one  


# OWL\_C.php

## Warnings:

  
**Warning on line 25** - no @package tag was used in a DocBlock for class OWL  
**Warning on line 312** - File "c:\cvs\rdfapi-php\api\vocabulary\OWL\_C.php" has no page-level DocBlock, use @package in the first DocBlock to create one  


# OWL\_RES.php

## Warnings:

  
**Warning on line 25** - no @package tag was used in a DocBlock for class OWL\_RES  
**Warning on line 312** - File "c:\cvs\rdfapi-php\api\vocabulary\OWL\_RES.php" has no page-level DocBlock, use @package in the first DocBlock to create one  


# Quad.php

## Warnings:

  
**Warning on line 20** - DocBlock would be page-level, but precedes class "Quad", use another DocBlock to document the file  

## Errors:

  
**Error on line 19** - unmatched ol or ul tag in DocBlock, parsing will be incorrect  
**Error on line 19** - Unclosed list tag in DocBlock, parsing will be incorrect  


# RDF.php

## Warnings:

  
**Warning on line 52** - File "c:\cvs\rdfapi-php\api\vocabulary\RDF.php" has no page-level DocBlock, use @package in the first DocBlock to create one  


# RdfAPI.php

## Warnings:

  
**Warning on line 30** - File "c:\cvs\rdfapi-php\api\RdfAPI.php" has no page-level DocBlock, use @package in the first DocBlock to create one  


# RdfParser.php

## Warnings:

  
**Warning on line 78** - DocBlock would be page-level, but precedes class "RdfParser", use another DocBlock to document the file  


# RDFS.php

## Warnings:

  
**Warning on line 38** - File "c:\cvs\rdfapi-php\api\vocabulary\RDFS.php" has no page-level DocBlock, use @package in the first DocBlock to create one  


# RdfSerializer.php

## Warnings:

  
**Warning on line 42** - DocBlock would be page-level, but precedes class "RdfSerializer", use another DocBlock to document the file  


# RDFS\_C.php

## Warnings:

  
**Warning on line 21** - no @package tag was used in a DocBlock for class RDFS  
**Warning on line 113** - File "c:\cvs\rdfapi-php\api\vocabulary\RDFS\_C.php" has no page-level DocBlock, use @package in the first DocBlock to create one  


# RDFS\_RES.php

## Warnings:

  
**Warning on line 22** - no @package tag was used in a DocBlock for class RDFS\_RES  
**Warning on line 113** - File "c:\cvs\rdfapi-php\api\vocabulary\RDFS\_RES.php" has no page-level DocBlock, use @package in the first DocBlock to create one  


# RdfUtil.php

## Warnings:

  
**Warning on line 29** - DocBlock would be page-level, but precedes class "RDFUtil", use another DocBlock to document the file  

## Errors:

  
**Error on line 29** - Text cannot be outside of li tag in a DocBlock list, parsing will be incorrect  


# RDF\_C.php

## Warnings:

  
**Warning on line 18** - no @package tag was used in a DocBlock for class RDF  
**Warning on line 184** - File "c:\cvs\rdfapi-php\api\vocabulary\RDF\_C.php" has no page-level DocBlock, use @package in the first DocBlock to create one  


# RDF\_RES.php

## Warnings:

  
**Warning on line 19** - no @package tag was used in a DocBlock for class RDF\_RES  
**Warning on line 185** - File "c:\cvs\rdfapi-php\api\vocabulary\RDF\_RES.php" has no page-level DocBlock, use @package in the first DocBlock to create one  


# RDQL.php

## Warnings:

  
**Warning on line 16** - File "c:\cvs\rdfapi-php\api\rdql\RDQL.php" has no page-level DocBlock, use @package in the first DocBlock to create one  


# RdqlDbEngine.php

## Warnings:

  
**Warning on line 31** - DocBlock would be page-level, but precedes class "RdqlDbEngine", use another DocBlock to document the file  

## Errors:

  
**Error on line 31** - unmatched ol or ul tag in DocBlock, parsing will be incorrect  
**Error on line 31** - Unclosed list tag in DocBlock, parsing will be incorrect  


# RdqlEngine.php

## Warnings:

  
**Warning on line 19** - DocBlock would be page-level, but precedes class "RdqlEngine", use another DocBlock to document the file  

## Errors:

  
**Error on line 19** - Unclosed list tag in DocBlock, parsing will be incorrect  
**Error on line 19** - unmatched ol or ul tag in DocBlock, parsing will be incorrect  


# RdqlMemEngine.php

## Warnings:

  
**Warning on line 35** - DocBlock would be page-level, but precedes class "RdqlMemEngine", use another DocBlock to document the file  

## Errors:

  
**Error on line 35** - unmatched ol or ul tag in DocBlock, parsing will be incorrect  
**Error on line 35** - Unclosed list tag in DocBlock, parsing will be incorrect  


# RdqlParser.php

## Warnings:

  
**Warning on line 34** - DocBlock would be page-level, but precedes class "RdqlParser", use another DocBlock to document the file  
**Warning on line 396** - Unknown tag "@toDo" used  

## Errors:

  
**Error on line 34** - unmatched ol or ul tag in DocBlock, parsing will be incorrect  
**Error on line 34** - Unclosed list tag in DocBlock, parsing will be incorrect  


# RdqlResultIterator.php

## Warnings:

  
**Warning on line 24** - DocBlock would be page-level, but precedes class "RdqlResultIterator", use another DocBlock to document the file  


# ResAlt.php

## Warnings:

  
**Warning on line 19** - DocBlock would be page-level, but precedes class "ResAlt", use another DocBlock to document the file  

## Errors:

  
**Error on line 18** - unmatched ol or ul tag in DocBlock, parsing will be incorrect  
**Error on line 18** - Unclosed list tag in DocBlock, parsing will be incorrect  


# ResBag.php

## Warnings:

  
**Warning on line 19** - DocBlock would be page-level, but precedes class "ResBag", use another DocBlock to document the file  

## Errors:

  
**Error on line 18** - unmatched ol or ul tag in DocBlock, parsing will be incorrect  
**Error on line 18** - Unclosed list tag in DocBlock, parsing will be incorrect  


# ResContainer.php

## Warnings:

  
**Warning on line 21** - DocBlock would be page-level, but precedes class "ResContainer", use another DocBlock to document the file  

## Errors:

  
**Error on line 20** - unmatched ol or ul tag in DocBlock, parsing will be incorrect  
**Error on line 20** - Unclosed list tag in DocBlock, parsing will be incorrect  


# ResList.php

## Warnings:

  
**Warning on line 32** - DocBlock would be page-level, but precedes class "ResList", use another DocBlock to document the file  

## Errors:

  
**Error on line 31** - unmatched ol or ul tag in DocBlock, parsing will be incorrect  
**Error on line 31** - Unclosed list tag in DocBlock, parsing will be incorrect  


# ResLiteral.php

## Warnings:

  
**Warning on line 23** - DocBlock would be page-level, but precedes class "ResLiteral", use another DocBlock to document the file  

## Errors:

  
**Error on line 22** - Unclosed list tag in DocBlock, parsing will be incorrect  
**Error on line 22** - unmatched ol or ul tag in DocBlock, parsing will be incorrect  


# ResModel.php

## Warnings:

  
**Warning on line 29** - DocBlock would be page-level, but precedes class "ResModel", use another DocBlock to document the file  
**Warning on line 663** - Unknown tag "@throw" used  


# ResModelP.php

## Warnings:

  
**Warning on line 26** - File "c:\cvs\rdfapi-php\api\resModel\ResModelP.php" has no page-level DocBlock, use @package in the first DocBlock to create one  


# Resource.php

## Warnings:

  
**Warning on line 29** - DocBlock would be page-level, but precedes class "Resource", use another DocBlock to document the file  


# ResProperty.php

## Warnings:

  
**Warning on line 19** - DocBlock would be page-level, but precedes class "ResProperty", use another DocBlock to document the file  

## Errors:

  
**Error on line 18** - unmatched ol or ul tag in DocBlock, parsing will be incorrect  
**Error on line 18** - Unclosed list tag in DocBlock, parsing will be incorrect  


# ResSeq.php

## Warnings:

  
**Warning on line 19** - DocBlock would be page-level, but precedes class "ResSeq", use another DocBlock to document the file  

## Errors:

  
**Error on line 18** - unmatched ol or ul tag in DocBlock, parsing will be incorrect  
**Error on line 18** - Unclosed list tag in DocBlock, parsing will be incorrect  


# RSS.php

## Warnings:

  
**Warning on line 37** - File "c:\cvs\rdfapi-php\api\vocabulary\RSS.php" has no page-level DocBlock, use @package in the first DocBlock to create one  


# RSS\_C.php

## Warnings:

  
**Warning on line 20** - no @package tag was used in a DocBlock for class RSS  
**Warning on line 84** - File "c:\cvs\rdfapi-php\api\vocabulary\RSS\_C.php" has no page-level DocBlock, use @package in the first DocBlock to create one  


# RSS\_RES.php

## Warnings:

  
**Warning on line 19** - no @package tag was used in a DocBlock for class RSS\_RES  
**Warning on line 82** - File "c:\cvs\rdfapi-php\api\vocabulary\RSS\_RES.php" has no page-level DocBlock, use @package in the first DocBlock to create one  


# Statement.php

## Warnings:

  
**Warning on line 21** - DocBlock would be page-level, but precedes class "Statement", use another DocBlock to document the file  

## Errors:

  
**Error on line 21** - Unclosed list tag in DocBlock, parsing will be incorrect  
**Error on line 21** - unmatched ol or ul tag in DocBlock, parsing will be incorrect  


# StatementIterator.php

## Warnings:

  
**Warning on line 25** - DocBlock would be page-level, but precedes class "StatementIterator", use another DocBlock to document the file  


# SyntaxGRDDL.php

## Warnings:

  
**Warning on line 17** - File "c:\cvs\rdfapi-php\api\syntax\SyntaxGRDDL.php" has no page-level DocBlock, use @package in the first DocBlock to create one  


# SyntaxN3.php

## Warnings:

  
**Warning on line 19** - File "c:\cvs\rdfapi-php\api\syntax\SyntaxN3.php" has no page-level DocBlock, use @package in the first DocBlock to create one  


# SyntaxRDF.php

## Warnings:

  
**Warning on line 19** - File "c:\cvs\rdfapi-php\api\syntax\SyntaxRDF.php" has no page-level DocBlock, use @package in the first DocBlock to create one  


# TriXParser.php

## Warnings:

  
**Warning on line 20** - DocBlock would be page-level, but precedes class "TriXParser", use another DocBlock to document the file  

## Errors:

  
**Error on line 19** - unmatched ol or ul tag in DocBlock, parsing will be incorrect  
**Error on line 19** - Unclosed list tag in DocBlock, parsing will be incorrect  


# TriXSerializer.php

## Warnings:

  
**Warning on line 19** - DocBlock would be page-level, but precedes class "TriXSerializer", use another DocBlock to document the file  

## Errors:

  
**Error on line 18** - Unclosed list tag in DocBlock, parsing will be incorrect  
**Error on line 18** - unmatched ol or ul tag in DocBlock, parsing will be incorrect  


# Utility.php

## Warnings:

  
**Warning on line 18** - File "c:\cvs\rdfapi-php\api\util\Utility.php" has no page-level DocBlock, use @package in the first DocBlock to create one  


# VCARD.php

## Warnings:

  
**Warning on line 71** - File "c:\cvs\rdfapi-php\api\vocabulary\VCARD.php" has no page-level DocBlock, use @package in the first DocBlock to create one  


# VCARD\_C.php

## Warnings:

  
**Warning on line 20** - no @package tag was used in a DocBlock for class VCARD  
**Warning on line 305** - File "c:\cvs\rdfapi-php\api\vocabulary\VCARD\_C.php" has no page-level DocBlock, use @package in the first DocBlock to create one  


# VCARD\_RES.php

## Warnings:

  
**Warning on line 20** - no @package tag was used in a DocBlock for class VCARD\_RES  
**Warning on line 306** - File "c:\cvs\rdfapi-php\api\vocabulary\VCARD\_RES.php" has no page-level DocBlock, use @package in the first DocBlock to create one  


# Vocabulary.php

## Warnings:

  
**Warning on line 23** - File "c:\cvs\rdfapi-php\api\vocabulary\Vocabulary.php" has no page-level DocBlock, use @package in the first DocBlock to create one  


# VocabularyClass.php

## Warnings:

  
**Warning on line 24** - File "c:\cvs\rdfapi-php\api\vocabulary\VocabularyClass.php" has no page-level DocBlock, use @package in the first DocBlock to create one  


# VocabularyRes.php

## Warnings:

  
**Warning on line 25** - File "c:\cvs\rdfapi-php\api\vocabulary\VocabularyRes.php" has no page-level DocBlock, use @package in the first DocBlock to create one  


# ResResource.php

## Errors:

  
**Error on line 24** - unmatched ol or ul tag in DocBlock, parsing will be incorrect  
**Error on line 24** - Unclosed list tag in DocBlock, parsing will be incorrect  

Documentation generated on Thu, 7 Jul 2005 13:44:01 +0200 by phpDocumentor 1.3.0RC3
